# Exploration of prognostic index based on immune-related genes in patients with liver hepatocellular carcinoma

**DOI:** 10.1042/BSR20194240

**Published:** 2020-06-30

**Authors:** Weidong Shi, Lanyun Feng, Shu Dong, Zhouyu Ning, Yongqiang Hua, Luming Liu, Zhen Chen, Zhiqiang Meng

**Affiliations:** 1 Department of Integrative Oncology, Fudan University Shanghai Cancer Center, Shanghai, China; 2 Department of Oncology, Shanghai Medical College, Fudan University, Shanghai, China; 3 Collaborative Innovation Center for Cancer Medicine, Fudan University Shanghai Cancer Center, Shanghai, China

**Keywords:** computation, immune-related genes, liver cancer, survival

## Abstract

The present study aimed to screen the immune-related genes (IRGs) in patients with liver hepatocellular carcinoma (LIHC) and construct a synthetic index for indicating the prognostic outcomes. The bioinformatic analysis was performed on the data of 374 cancer tissues and 50 normal tissues, which were downloaded from TCGA database. We observed that 17 differentially expressed IRGs were significantly associated with survival in LIHC patients. These LIHC-specific IRGs were validated with function analysis and molecular characteristics. Cox analysis was applied for constructing a RiskScore for predicting the survival. The RiskScore involved six IRGs and corresponding coefficients, which was calculated with the following formula: RiskScore = [Expression level of FABP5 *(0.064)] + [Expression level of TRAF3 * (0.198)] + [Expression level of CSPG5 * (0.416)] + [Expression level of IL17D * (0.197)] + [Expression level of STC2 * (0.036)] + [Expression level of BRD8 * (0.140)]. The RiskScore was positively associated with the poor survival, which was verified with the dataset from ICGC database. Further analysis revealed that the RiskScore was independent of any other clinical feature, while it was linked with the infiltration levels of six types of immune cells. Our study reported the survival-associated IRGs in LIHC and then constructed IRGs-based RiskScore as prognostic indicator for screening patients with high risk of short survival. Both the screened IRGs and IRGs-based RiskScore were clinically significant, which may be informative for promoting the individualized immunotherapy against LIHC.

## Introduction

Liver cancer has become the second leading cause of cancer-related mortality, leading to 745,000 deaths every year in the world. The highest incidence of liver cancer has been observed in developing countries, due to the relatively poor economic conditions and living environment [1]. The hepatitis B and C virus infection, drinking, smoking and aflatoxin exposure were all common risk factors of liver cancer [2]. Chinese population show a high risk of liver cancer because of the high prevalence of hepatitis B virus in the world, up to nearly 10% of the general population [3]. One study predicted that the incidence rate of liver cancer was 40.0 (in males) and 15.3 (in females) per 100,000 world standard population. Such a high incidence rate led to heavy economic and social burden to the country [2]. Despite of technic advances, the treatment of liver cancer was still an important health issue for the unfavorable prognostic outcomes. However, the precise early diagnosis facilitated more and better treatment options [4].

Among the therapy strategies of liver cancer, immunotherapy has been one of the most significant drivers of personalized medicine, providing good efficacy and favorable outcomes [5,6]. The immune system of patients has been mobilized to fight against the tumors. The liver is a central immunomodulator for providing systemic protection and maintaining immunotolerance. The dysregulation of liver-based immunological network and circulation has been associated with the pathogenesis of hepatocellular diseases, finally leading to liver cancer [7]. Several innovative immunotherapies have been developed for the treatment of liver cancer, such as diverse vaccine platforms, adoptive T-cell therapy, cytokines, gene therapy and monoclonal antibodies that targeted immune checkpoint molecules. We noted that some of them exhibited good efficacy [5]*.* Among these therapies, the immunostimulatory cytokine-targeted monoclonal antibodies, adoptive T-cell therapy or vaccines in combination with gene therapy have been confirmed as powerful weapon for liver cancer [8]*.* The survival benefits of these agents have been demonstrated in both clinical practices and phase II/III studies, such as sorafenib and lenvatinib [8].

In-depth exploration of genetic characteristics of patients with liver cancer presents following benefits: identifying the patients responsive or resistant to a certain immunotherapy; optimizing the combinations of molecularly targeted therapies; predicting the clinical features, outcome and progression of diseases. Many reviews have summarized the significant targets in the liver cancer, which can be applied in both therapy and diagnosis, allowing a better patient selection for personalized medicine [9]. However, more such significant biomarker candidates should be screened and verified in the clinical practices, which may assist in developing better therapy in the future [10].

Immune-related genes (IRGs) have been proved to play key roles in the regulation of systemic immune response. Learning more about the IRGs has been essential to demonstrate the mechanism of immunotherapy against cancer. Some critical IRGs could be promising biomarkers for predicting the outcome of cancer after the treatment [11,12]. Our study is aimed to analyze survival-associated IRGs in liver hepatocellular carcinoma (LIHC). We first identified differentially expressed IRGs between normal and cancerous tissues; then, the IRGs associated with prognostic outcomes were screened; third, a RiskScore was constructed with these IRGs, which can be applied as a predictor for distinguishing the LIHC patients with high risk of poor prognosis; finally, the clinical significance of RiskScore was systematically validated. The promising results from the present study could offer information for following, in-depth immune-related work for personalized immunotherapy against LIHC.

## Materials and methods

### Clinical data acquisition and extraction

Transcriptome RNA-sequencing data of LIHC were downloaded from the TCGA data portal (https://cancergenome.nih.gov/). The Fragments per Kilobase Million (FPKM) expression profiling data were applied for analysis. There were 374 cases of LIHC tissues and 50 cases of normal tissues. The clinical information and demographic data were also obtained from the TCGA data portal. The list of IRGs was obtained from Immunology Database and Analysis Portal (ImmPort) database [13]. The IRGs in the list were identified as critical genes involved in the immune activity. A total of 2498 IRGs were considered.

### Differential expressed genes (DEGs) and differentially expressed IRGs

Differential expressed genes (DEGs) between LIHC tissues and normal tissues were preliminarily screened via the R software Limma package. The data were presented with heatmap and volcano plot with R software Pheatmap package. DEGs were determined with the following cutoff value: false discovery rate (FDR) = 0.05, log2 |fold change| = 1. The differential gene analysis was performed with wilcox test. Differentially expressed IRGs were then extracted from all screened DEGs according to the IRGs list. Gene functional analyses were performed via the Gene ontology (GO) and Kyoto Encyclopedia of Genes and Genomes (KEGG) pathways enrichments with R software Clusterprofiler package.

### Survival analysis

The overall survival was involved as the endpoint and index for the prognostic outcome. The clinical follow-up information was obtained from TCGA’s Pan-Cancer Atlas (https://cancergenome.nih.gov/). All the data were randomly and equally divided into two groups (Train group and Test group) with caret R package. The data in Train group were applied for further analysis, while the data in Test group were applied for validation.

For the Train group, the survival R package was applied to screen the survival-associated IRGs by univariate Cox analysis, with *P*<0.001. Only the cases with survival interval longer than 90 days were included in the analysis. The hazard ratio (HR) was calculated and expressed with forest plot. GO enrichment was performed to explore potential molecular mechanisms of the significant survival-associated IRGs.

### The transcription factors (TFs) regulatory network

Transcription factors (TFs) were important molecules that directly controlled the levels of gene expression. The Cistrome Cancer database was a valuable resource for experimental and computational cancer biology research and contained 318 TFs (http://cistrome.org/). The differentially expressed TFs were screened with the following cutoff value: false discovery rate (FDR) = 0.05, log2 |fold change| = 1. A total of 117 differentially expressed TFs were screened. The Cytoscape (v3.6.1) was applied to construct the regulatory network of the survival-associated IRGs and differentially expressed TFs.

### The construction of IRGs-based RiskScore

For the Train group, multivariate Cox analysis was performed to screen the survival-associated IRGs with survival R package (with *P*<0.001). In the optimized model, the included IRGs were presented, with corresponding coefficients and HRs.

For the IRGs included in the RiskScore, the gene set enrichment analysis (GSEA) was applied to enrich these IRGs to identify their potential functions. Copy number alterations data were obtained from Cbioportal (http://www.cbioportal.org/) and applied to evaluate the molecular characteristics of the included IRGs.

The RiskScore was calculated based on the expression level of included IRGs and their corresponding coefficient, which was obtained in multivariate Cox analysis. With the RiskScore, patients were divided into high- and low-risk groups. The prognostic prediction value of RiskScore was evaluated in the two groups. Then, the ROC curve was plotted. AUC of the survival ROC curve was calculated via the survival ROC R software package.

### The validation of RiskScore as prognostic index

The RiskScore was validated with the cases in Test group, with the same method as in Train group. In addition, the RiskScore was validated in another dataset downloaded from ICGC database. The dataset was derived from Project LIRI-JP, including 243 LIHC samples. The receiver operating characteristic (ROC) curve was plotted and the area under curve of ROC (AUC) was calculated.

### The relationship of RiskScore with clinical and demographic characteristics

**Scheme 1:**
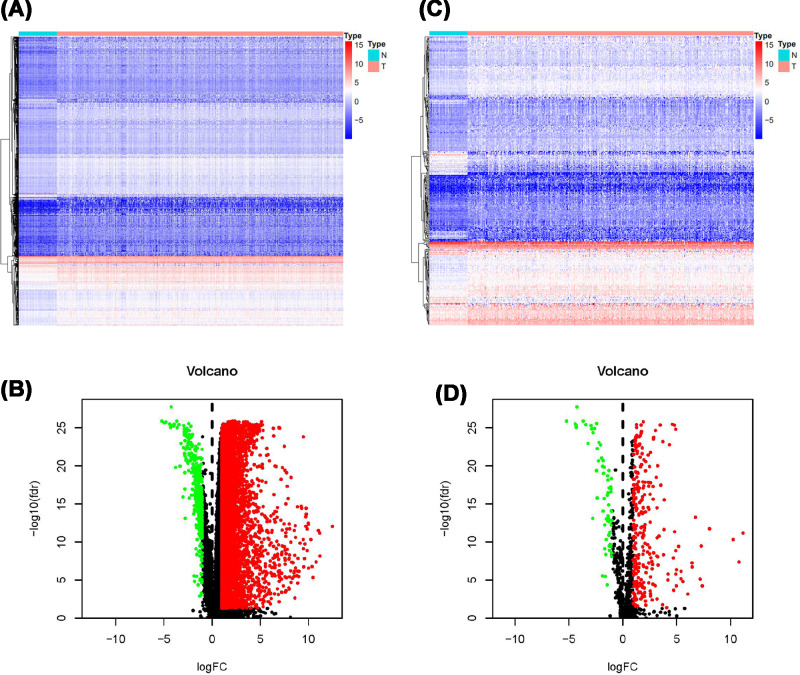
Flow diagram of the analysis procedure: data collection, preprocessing, analysis and validation

The univariate and multivariate Cox analysis was applied to evaluate the prognostic value of RiskScore. The beeswarm R package and *T* test were involved to explore the relationships between the IRGs-based RiskScore and clinical and demographic characteristics, including age, gender, grade, American Joint Committee on Cancer (AJCC) stage and TNM (Tumor, Node, Metastasis). *P*<0.05 indicated statistically significant (Scheme 1).

### The relationship of RiskScore with tumor infiltrating immune cells

The immune infiltration levels of LIHC patients were downloaded and the abundance of six subtypes of tumor-infiltrating immune cells included B cells, CD4 T cells, CD8 T cells, macrophages, neutrophils and dendritic cells. The TIMER online database analyzed and visualized the abundance of tumor infiltrating immune cells (https://cistrome.shinyapps.io/timer/). The associations between the RiskScore and immune cells infiltration were analyzed; *P*<0.05 indicated statistically significant.

## Results

### Identification of DEGs and IRGs

**Figure 1:**
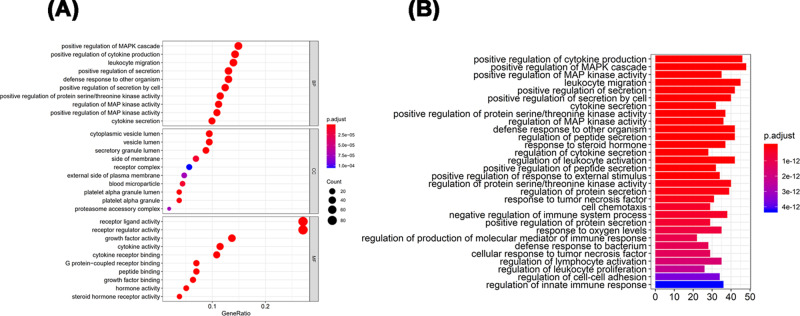
DEGs and IRGs (**A**) Heatmap and (**B**) volcano plot of DEGs between normal and LIHC tissues. (**C**) Heatmap and (**D**) volcano plot of differentially expressed IRGs. Notes: in (A) and (C), blue dots represent differentially expressed genes and red dots represent not differentially expressed genes.

The dataset was downloaded from TCGA data portal. There were 374 cases of LIHC tissues and 50 cases of normal tissues. A total of 7667 DEGs between LIHC and normal tissues were identified, including 7273 up-regulated and 394 down-regulated DEGs (Figure 1A,B). Then, the IRGs were exacted according to the IRGs list from ImmPort database. Among these DEGs, we extracted 329 differentially expressed IRGs, including 267 up-regulated and 62 down-regulated (Figure 1C,D).

**Figure 2:**
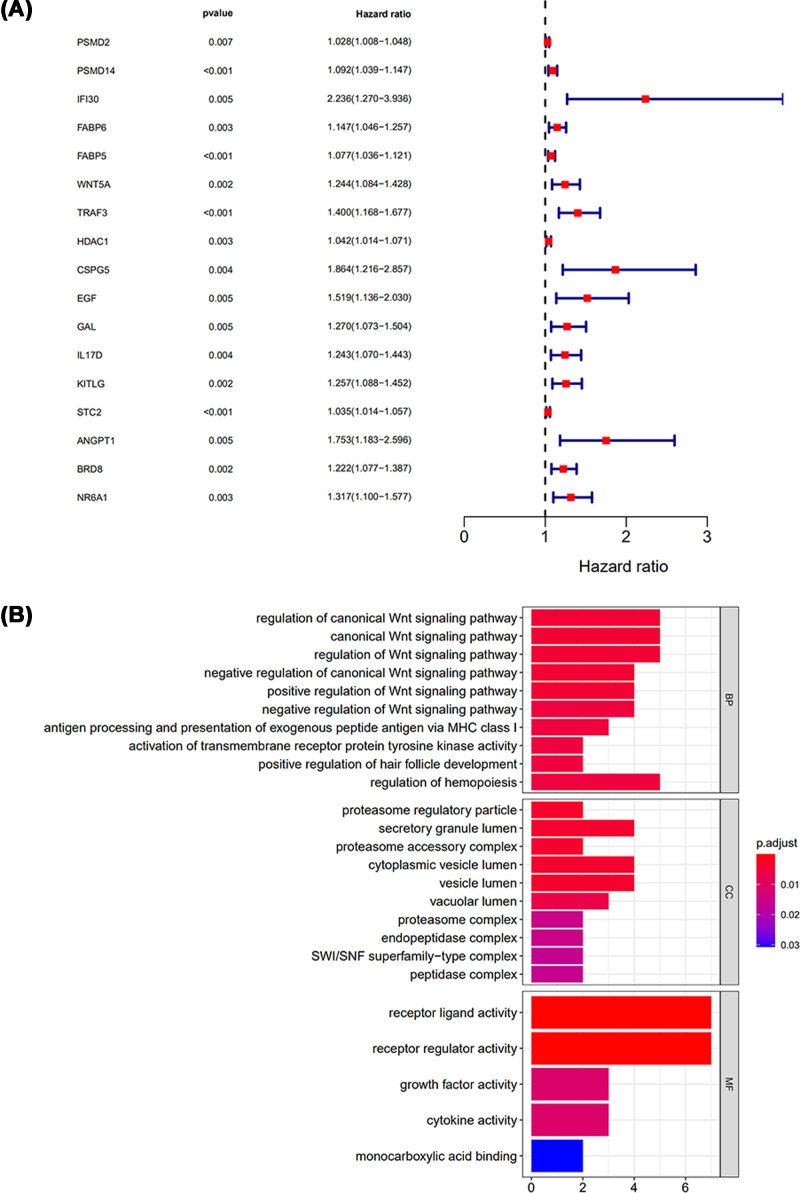
Gene functional enrichment of differentially expressed IRGs (**A**) GO analysis: BP represent biological process, CC indicated cellular component and MF represented molecular function, respectively. (**B**) KEGG pathways analysis.

Gene functional enrichment was performed on these differentially expressed IRGs. From the GO analysis, “positive regulation of MARP cascade”, “cytoplasmic vesicle lumen” and “receptor ligand activity” were the most frequent biological terms among biological processes, cellular components and molecular functions, respectively (Figure 2A). For the KEGG pathways, “positive regulation of cytokine production” was most often enriched (Figure 2B).

### Survival-associated IRGs

**Figure 3:**
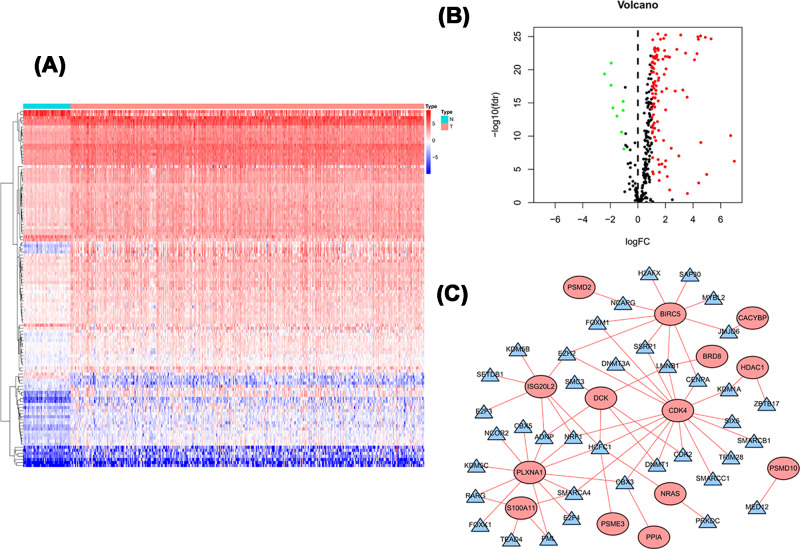
Identification of survival-associated differentially expressed IRGs (**A**) Forest plot of hazard ratios presented the prognostic values of IRGs. (**B**) KEGG pathway analysis of survival-associated IRGs.

Considering the high mortality of LIHC, survival was the most significant index for indicating the prognostic outcomes of LIHC patients. The potential relationship between IRGs and survival was analyzed with R survival package. The results suggested that 17 IRGs were significantly correlated to the survival in LIHC patients. The HRs were presented with forest plot (Figure 3A).

The 17 survival-associated IRGs were analyzed with GO analysis. Similar to the GO analysis result of all differentially expressed IRGs, “receptor ligand activity” was the most enriched molecular function. Besides, “regulation of canonical Wnt signaling pathway”, “secretory granule lumen” and “receptor ligand activity” were the most frequently enriched biological processes and cellular components, respectively (Figure 3B).

### The TF regulatory network

**Figure 4:**
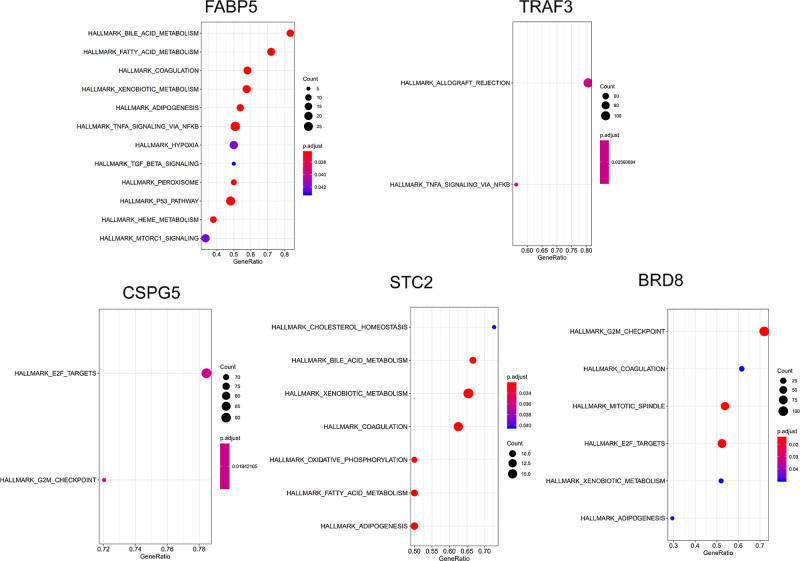
TF-mediated regulatory network (**A**) Heatmap and (**B**) volcano plot of differentially expressed TFs. (**C**) Regulatory network constructed based on differentially expressed TFs and survival-associated IRGs. Notes: blue triangle indicated TFs; red circle indicated IRGs.

The TF regulatory network was investigated to explore the potential molecular mechanisms of the screened survival-associated IRGs. The expression profiles of 318 TFs were analyzed between 374 cases of LIHC and 50 cases of normal tissues. A total of 117 differentially expressed TFs were obtained (Figure 4A,B). Then, a regulatory network was constructed based on above 117 TFs and the 17 IRGs. A correlation score more than 0.6 and *P*-value less than 0.001 were set as the cut-off. The TF-based regulatory network presented the regulatory relationships among these IRGs (Figure 4C).

### The construction of RiskScore as a prognostic index

All the sample were randomly and equally divided into Train and Test groups with caret R package. For the Train group, the multivariate Cox regression analysis was performed to construct a RiskScore to classify the LIHC patients into two groups with different survival. The coefficients and HRs of IRGs optimized in multivariate Cox regression analysis were presented (Table 1). The RiskScore can be calculated with the following formula: RiskScore = [Expression level of FABP5 *(0.064)] + [Expression level of TRAF3 * (0.198)] + [Expression level of CSPG5 * (0.416)] + [Expression level of IL17D * (0.197)] + [Expression level of STC2 * (0.036)] + [Expression level of BRD8 * (0.140)].

**Table 1:** The results of Cox regression analysis

IRGs	coef	HR	HR.95L	HR.95H	*P* value
FABP5	0.064	1.066	1.017	1.117	0.007
TRAF3	0.198	1.219	0.978	1.519	0.079
CSPG5	0.416	1.515	0.929	2.473	0.096
IL17D	0.197	1.217	1.037	1.429	0.016
STC2	0.036	1.037	1.012	1.062	0.003
BRD8	0.140	1.150	0.994	1.330	0.060

**Figure 5:**
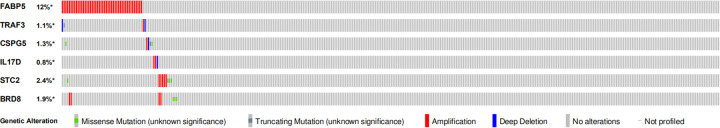
Gene Set Enrichment Analysis of survival-associated differentially expressed IRGs Notes: for IL17D, GSEA no term enriched under specific *P* value cutoff.

Six IRGs were included in the calculation formula of RiskScore. These IRGs were further analyzed with GSEA except for IL17D (no term enriched under specific *P* value cutoff). To identify the potential functions of the optimized IRGs in LIHC, GSEA was performed to search KEGG pathways enriched with single highly-expressed gene. From the results of GSEA, “bile-acid-metabolism” was enriched for FABP5. “allograft rejection” was enriched for TRAF3. “cholesterol homeostasis” was enriched for STC2 (Figure 5). All these biological terms were associated with liver functions.

**Figure 6:**
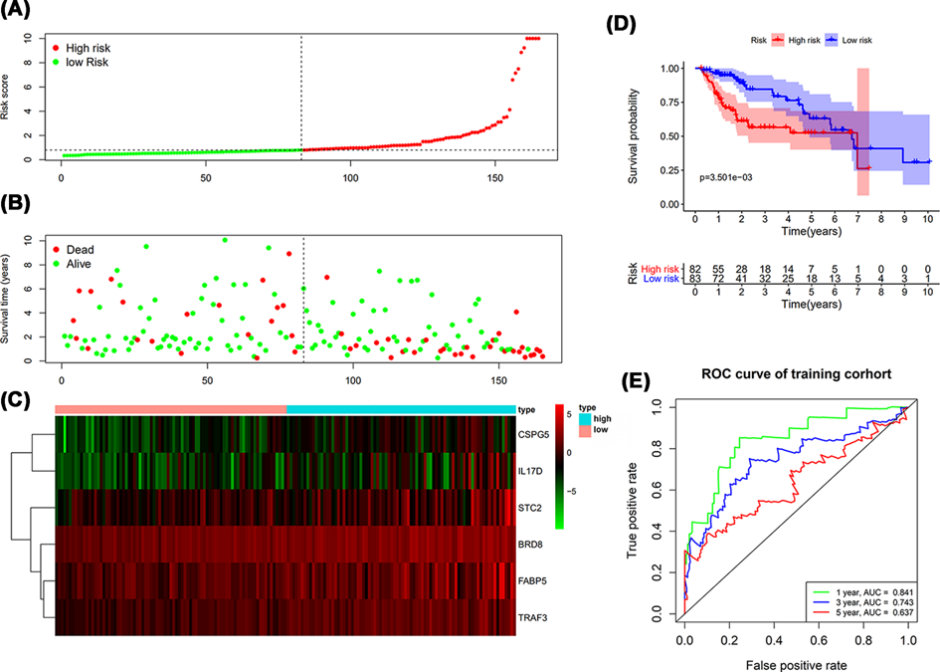
Mutation landscape of survival-associated differentially expressed IRGs FABP5 was the gene with the highest mutation frequency up to 12%.

The molecular characteristics were further explored for the involved six IRGs. We examined genetic alterations of these genes and found that FABP5 showed the highest mutation rate (12%). In addition, amplification and deep deletion were the two most commonly occurring types of mutation (Figure 6).

### The prognostic prediction of RiskScore

**Figure 7:**
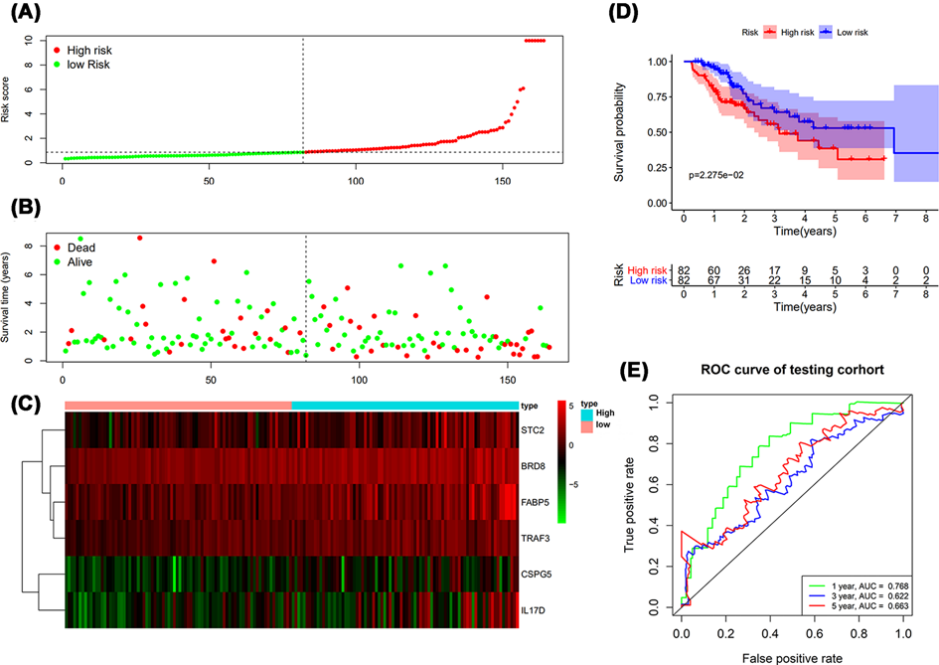
The prognostic results of IRGs-based RiskScore in Train group (**A**) Rank of prognostic index and distribution of high and low risk groups. (**B**) Survival status of patients in different groups. (**C**) Heatmap of expression profiles of included genes in high and low risk groups. (**D**) Patients in high-risk group showed shorter survival intervals. (**E**) Survival-dependent receiver operating characteristic (ROC) curve indicated prognostic results in train group. The area under curve (AUC) corresponding to 1-, 3- and 5-year survival was provided.

The clinical significance of IRGs-based RiskScore was evaluated and validated with patients in Train group, which can be applied for predicting the survival of LIHC patients. With the RiskScore, the LIHC patients can be separated into two groups with different survival (Figure 7A–C). The survival intervals could be significantly differentiated in LIHC patients with high and low risk (Figure 7D). Survival-dependent ROC curve was plotted and the AUC was obtained, which was 0.841, 0.743 and 0.637 for the 1-, 3- and 5-year survival. The results suggested moderate potential for the IRGs based RiskScore in survival monitor and prediction (Figure 7E).

### The validation of prognostic value of RiskScore

**Figure 8:**
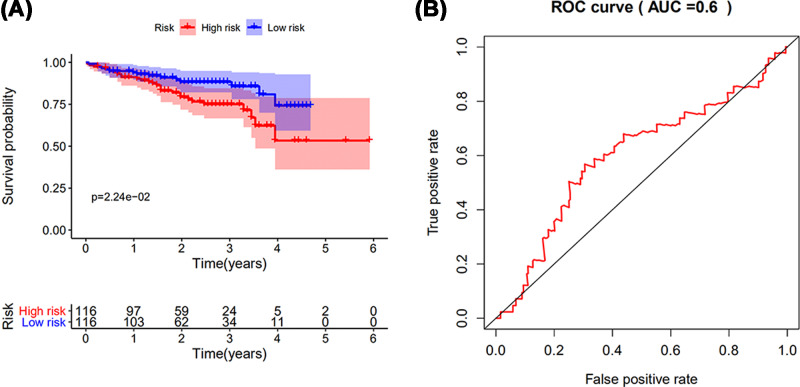
The validation of prognostic results in Test group (**A**) Rank of prognostic index and distribution of high and low risk groups. (**B**) Survival status of patients in different groups. (**C**) Heatmap of expression profiles of included genes in high and low risk groups. (**D**) Patients in high-risk group showed shorter survival intervals. (**E**) Survival-dependent ROC curve indicated prognostic results in test group. The AUC corresponding to 1-, 3- and 5-year survival was provided.

The RiskScore based prognostic prediction index was validated in Test group. With the RiskScore calculated from the Train group, the LIHC patients could also be separated into two groups, the high risk and low risk group (Figure 8A–C). The survival intervals could be significantly differentiated in LIHC patients from two groups (Figure 8D), with an AUC of 0.768, 0.622 and 0.663 corresponding to the 1-, 3- and 5-year survival. The results of Test group validated that RiskScore obtained in Train group was also significant in survival monitor and prediction (Figure 8E).

**Figure 9:**
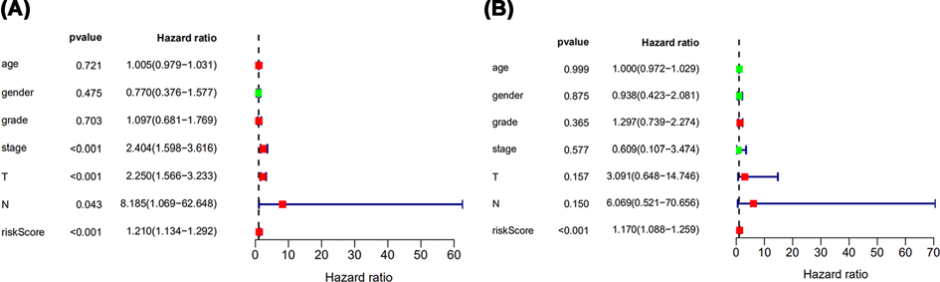
The validation of prognostic results in dataset from ICGC database (**A**) Patients in high-risk group suffered shorter survival intervals. (**B**) Survival-dependent ROC curve validation of prognostic results. The AUC was corresponding to 3-year survival.

The result was validated in dataset from ICGC database (Project LIRI-JP), including 243 LIHC samples. Patients in high-risk group suffered shorter survival intervals (Figure 9A). Survival-dependent ROC validated prognostic results, with the AUC of 0.6 for 3-year survival (Figure 9B).

**Figure 10:**
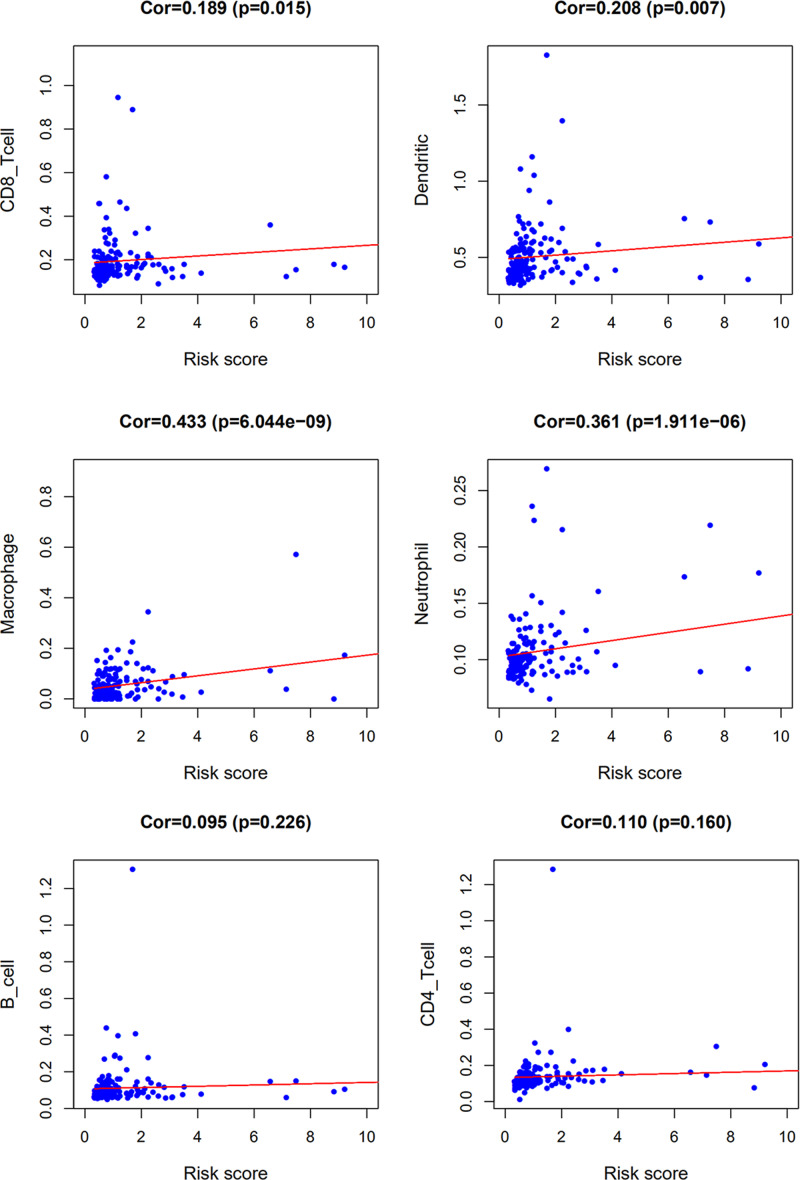
Independent prognostic analysis The univariate Cox analysis (**A**) and multivariate Cox analysis (**B**) for evaluating the hazard ratios of RiskScore in predicting the clinical and demographic characteristics, including age, gender, grade, AJCC stage, and TNM.

Relationships were analyzed between the IRGs-based RiskScore with clinical and demographic characteristics, including age, gender, grade, AJCC stage and TNM. The RiskScore seemed to be not correlated with any clinical and demographic characteristics (Table 2). The prognostic value of RiskScore was further evaluated with univariate Cox analysis (Figure 10A) and multivariate Cox analysis (Figure 10B). Both the results indicated that RiskScore could be a survival prediction index, independent of above clinical and demographic characteristics.

**Table 2:** Relationships between the expressions of each IRG with the clinicopathological factors in LIHC

IRGs	Age		Gender		Grade		Stage		*T*	
	*t*	*P*	*t*	*P*	*t*	*P*	*t*	*P*	*t*	*P*
FABP5	2.313	0.023	0.249	0.804	−0.623	0.535	−0.32	0.75	−0.32	0.75
TRAF3	1.393	0.167	0.168	0.867	−0.089	0.929	−1.928	0.064	−1.928	0.064
CSPG5	0.039	0.969	0.207	0.837	−1.787	0.078	−0.015	0.988	−0.015	0.988
IL17D	0.491	0.625	1.418	0.165	−1.416	0.161	−0.418	0.678	−0.418	0.678
STC2	−1.369	0.18	−1.441	0.153	−0.774	0.442	−1.28	0.212	−1.28	0.212
BRD8	0.29	0.773	2.239	0.031	−0.536	0.593	−2.171	0.035	−2.171	0.035
RiskScore	−0.781	0.44	0.389	0.698	−1.086	0.28	−1.542	0.135	−1.542	0.135

**Figure 11:**
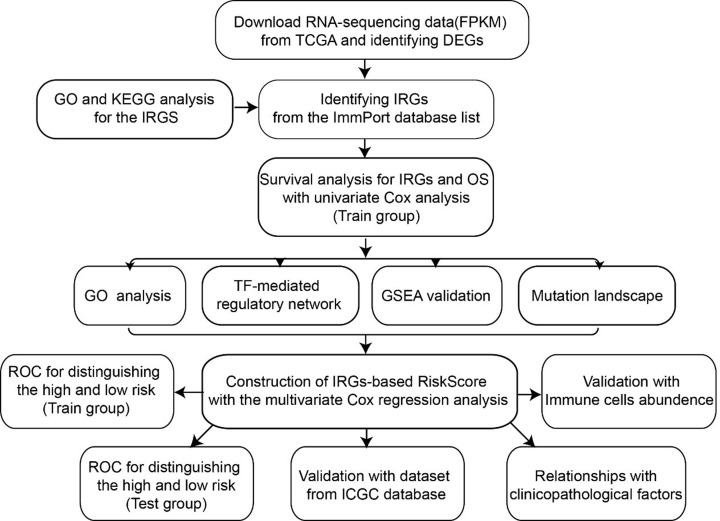
Relationships between the RiskScore and infiltration abundances of six types of immune cells The correlation was performed with Pearson correlation analysis.

Relationships were also explored between the IRGs-based RiskScore and tumor immune microenvironment. We analyzed the association between RiskScore and immune cell infiltration. The count of CD8_Tcell, Dendritic cell, Macrophage and Neutrophil was positively correlated with the RiskScore (Figure 11).

## Discussion

Immunotherapies have become promising individualized therapies against various cancers. However, the selection of appropriate therapy combination for individual patient has been one of the most critical issues [14]. Immunotherapy for LIHC has been well developed [10,15]. It provided innovative treatment options besides of sorafenib and other newly approved tyrosine kinase inhibitors. Immunotherapy for LIHC has been promising for its safety and efficacy. The progression of tumor can be delayed, especially for patients with advanced stages of liver cancers [15].

In September 2017, FDA approved nivolumab as a second line treatment for liver cancer after the failure of sorafenib. In the phase I/II trial, nivolumab showed response across all cohorts in 14–20% of patients. However, the main side adverse events were immune-related side effects [14]. Choosing the right patients for immunotherapy and predicting the prognostic outcomes has been essential. In previous studies, several indicators have been developed to evaluate the applicability of therapies on certain patients, as well as predicting the prognostic outcomes [12,16]. The IRGs have played critical roles in the tumorgenesis and progression, which were also believed to function in immunotherapies [11]. However, until now, limited studies have focused on their clinical significance and molecular mechanism.

Immune tolerance and suppression in tumor microenvironment was considered as the theoretical basis of immunotherapy [17]. Previous studies have ever analyzed the variations of microenvironments of LIHC from the perspectives of IRGs. One recent study identified a IRGs expression pattern in liver tissues of patients with early-stage hepatocellular carcinoma, and proved its association with the risk of cirrhosis. A total of 172 genes were involved in this IRGs expression signature [18]. Another experimental study revealed the relationship between expression levels of programmed cell death ligands (PD-Ls) and IRGs. The level of PD-Ls was a prediction indicator for clinical response to anti-PD-1 axis immunotherapy, and higher expression of IRGs was correlated with higher levels of PD-Ls [19].

Some IRGs were screened and elucidated. The TP53 has been strongly related to the immune microenvironment in liver cancer, which made effects in the cancer progression. The immune prognostic model dominated by TP53 mutation status was able to identify patients with low or high risk of unfavorable survival [20]. TSA regulated the transcription of numerous innate immunity and tumor antigen recognition-associated genes in hepatocellular carcinoma cells. Both the *in vivo* and *in vitro* results validated that TSA slowed the tumor cell growth via NK cell-mediated pathways. TSA also induced direct killing of hepatocellular carcinoma cells by stimulating apoptosis [21]. In our study, the differentially expressed IRGs in LIHC with clinical significance were screened and validated with bioinformatic approaches. A synthetical IRGs-based RiskScore was constructed for evaluating the prognostic outcome of patients with LIHC. The potential molecular characteristics were further elucidated.

After the IRGs were screened, the gene functional enrichment analysis was performed to explore the biological functions [22]. In our study, the preliminarily screened IRGs were mainly involved in “positive regulation of MARP cascade”, “cytoplasmic vesicle lumen” and “receptor ligand activity” corresponding to biological processes, cellular components and molecular functions, respectively. Further, the IRGs frequently participated in the pathways of positive regulation of cytokine production. Since we focused on the IRGs associated with the prognostic outcomes, the survival-associated IRGs were screened. For these survival-associated IRGs, “regulation of canonical Wnt signaling pathway”, “secretory granule lumen” and “receptor ligand activity” were the most frequent biological terms among biological processes, cellular components and molecular functions, respectively. TF-mediated regulatory network was also constructed to explore underlying molecular mechanisms [23]. Vital TFs that could regulate identified survival-associated IRGs were screened. In the 17 differentially expressed IRGs, 5 IRGs featured prominently in this network, including HDAC1, PSMD2, IFI30, TRAF3 and KITLG. Above results provided information for the mechanism exploration, which may ground the experimental validation.

The multivariate Cox regression analysis was applied to construct the RiskScore for predicting the survival of LIHC patients. In the optimized model, six differentially expressed IRGs were involved, including FABP5, BRD8, IL-17D, STC2, TRAF3 and CSPG5. All these genes were associated with the specific physiological functions. Some of them have been reported in previous studies on their roles in the initiation and progression of cancer.

Fatty acid binding proteins (FABPs) were a group of lipid binding proteins modulate fatty acid metabolism, cell growth and proliferation and cancer development. FABPs were over-expressed in many malignancies including prostate, breast, liver, bladder and lung cancers. They were associated with the incidence, proliferation, metastasis, invasion of tumors [24,25]. The overexpression of FABP5 was also reported in many types of tumor. In addition, up-regulation of FABP5 was strongly associated with poor survival in triple-negative breast cancer [26]. The mechanisms underlying the specific up-regulation of the FABP5 in these cancers remained poorly characterized. One study stated the overexpression of FABP5 in prostate cancer cells can be attributed to hypomethylation of the CpG island in its promoter region, along with up-regulation of the direct trans-acting factors Sp1 (specificity protein 1) and c-Myc [27]. An experimental study performed on mouse xenografts suggested that atelocollagen-delivered siRNA targeting the FABP5 gene could be applied as the therapy for prostate cancer [28]. Similar results have been obtained in human cells. The silence of FABP5 gene made effects on the proliferation, apoptosis and invasion of human gastric SGC-7901 cancer cells [29]. Above results from previous studies supported that the IRGs screened with our method showed clinical significance.

Stanniocalcin 2 (STC2) encoded a secreted, homodimeric glycoprotein that was widely expressed in various tissues, showing autocrine or paracrine functions. The prognostic prediction significance of STC2 has been confirmed in gastric cancer [30], breast cancer [31], colorectal cancer [32] and ovarian cancer [33]. The STC2 also played critical roles in LIHC. STC2 was up-regulated in hepatocellular carcinoma, promoting cell proliferation and migration *in vitro*. STC2 protein was a potential oncoprotein in the development and progression of liver cancer, thus being considered as a promising biomarker and molecular target for the anti-cancer therapy [34]. The action mechanism of STC2 in the cancer has been explored. STC2 overexpression in hepatocellular carcinoma could lead to poor prognosis, which might be due to its induced increase of P-glycoprotein and Bcl-2 protein expression levels. P-glycoprotein regulated drug efflux and Bcl-2 modulated drug resistance, both of which led to the failure of chemotherapy in hepatocellular carcinoma [35]. The roles played by STC2 involved the PI3K/AKT/Snail signaling and AKT-ERK signaling pathways [36,37].

Interleukin 17 (IL-17) was a pro-inflammatory cytokine mainly produced by activated T cells [38,39]. Most studies on IL-17 were associated with inflammation, while relatively fewer studies reported the effects of IL-17 in the cancers. A recent study summarized the roles of IL-17 in pathogenesis of colorectal cancer [40]. The prognostic significance of IL-17 was confirmed in patients with non-small-cell lung cancer (NSCLC), and the increased IL-17-producing cells was correlated with poor survival and lymphangiogenesis. The study revealed that IL-17 expression was an independent prognostic indicator for both overall and disease-free survival in NSCLC [41]. Until now, no study reported the association between IL-17 and LIHC. Only one recent study mentioned that IL-17 may contribute to autoimmune hepatitis [42].

Bromodomain 8 (BRD8) was an accessory subunit of human NuA4-HAT (histone acetyl transferase) complex, which was associated with chromatin regulation. A few studies have reported the roles of BRD8 in cancer. One study reported that BRD8 was involved in cellular survival, as well as sensitivity to spindle poisons and proteasome inhibitor in aggressive colorectal cancers. Targeting BRD8 would improve therapeutic outcome against aggressive/metastatic colorectal cancers. However, our study has been the first one to suggest the potential effects of BRD8 in LIHC.

Tumor necrosis factor receptor–associated factor 3 (TRAF3) regulated both innate and adaptive immunity by modulating signaling by Toll-like receptors (TLR) and TNF receptors. TRAF3 was recently identified as a tumor suppressor in human multiple myeloma. TRAF3 transgenic mice with overexpressed TRAF3 showed improved humoral responses, resulted in a series of symptoms including plasmacytosis, autoimmunity, inflammation and cancer [43]. The TRAF3 silenced by miR-214 contributed to the osteolytic bone metastasis of breast cancer [44]. Inhibition of osteoclastic miR-214-3p could be a promising therapeutic strategy for breast cancer patients with osteolytic bone metastasis. Meanwhile, the intraosseous TRAF3 could be a promising biomarker for evaluating the treatment response of antagomir-214-3p [44]. Another inspiring study explored that hepatocyte TRAF3 promoted liver steatosis and systemic insulin resistance through targeting TAK1-dependent signaling. Above results indicated that TRAF3 could be a promising factor in LIHC [45].

Chondroitin sulfate proteoglycans (CSPGs) were proteoglycans consisting of a core protein and chondroitin sulfate. Chondroitin sulfate proteoglycan 5 (CSPG5) gene encoded chondroitin GSPG5 in humans. Only one study stated the CSPG5 as a prognostic factor for breast cancer based on immunohistochemical analysis [46].

The IRGs-based RiskScore developed in our study were composed of above six screened IRGs with respective coefficient. The patients with higher RiskScore presented with shorter survival. For the six involved genes including FABP5, BRD8, IL-17D, STC2, TRAF3 and CSPG5, the formula was as follows: RiskScore = [Expression level of FABP5 *(0.064)] + [Expression level of TRAF3 * (0.198)] + [Expression level of CSPG5 * (0.416)] + [Expression level of IL17D * (0.197)] + [Expression level of STC2 * (0.036)] + [Expression level of BRD8 * (0.140)]. All the coefficients were positive, indicating all these IRGs were positively related to the risk of poor outcomes. It was consistent with the results from existing studies. Comparing with previous publications, the present study proposed a synthetical indicator for predicting the outcome with overall survival as the endpoint, which was the most suitable endpoint for monitoring the survival of LIHC patients. Furthermore, the IRG could be applied not only as a prognostic indicator, but also as an indicator for immune status. Our IRGs-based RiskScore demonstrated favorable clinical significance in LIHC.

Notably, the RiskScore obtained in our study was not associated with any other clinical and demographic characteristics, including age, gender, grade, AJCC stage and TNM. However, the RiskScore was positively correlated with the count of CD8_Tcell, Dendritic cell, Macrophage and Neutrophil. Therefore, the treatment could be adjusted according to the infiltration levels of immune cells.

However, there were some limitations in present study. First, the result was only validated with one dataset from another cohort. Second, the reliability of our results should be further proved with *in vitro* or *in vivo* experiments.

In conclusion, the data from 374 cancer tissues and 50 normal tissues were gathered and analyzed to obtain the differentially expressed IRGs. The survival associated IRGs were further screened and validated from various perspectives, including function analysis, molecular characteristics and so on. Bioinformatic analysis enabled a more in-depth exploration of molecular mechanisms. Then, a RiskScore was constructed with the Cox analysis, including six survival-associated IRGs and corresponding coefficient. The RiskScore was proved as a valuable prediction index for the prognosis of LIHC. The significance of the present study was as follows: (1) first reported the survival associated IRGs and screened six IRGs with clinical significance in LIHC based on bioinformatics; (2) constructed IRGs-based RiskScore as prognostic indicator for screening patients with high risk; (3) enhanced the understanding of immune-associated molecular mechanism of LIHC.

## Abbreviations

BRD8: bromodomain 8
CSPG: chondroitin sulfate proteoglycan
IRG: immune-related gene
LIHC: liver hepatocellular carcinoma
TLR: Toll-like receptors
TRAF3: tumor necrosis factor receptor–associated factor 3
